# Relapsing Fever in Animals

**Published:** 1886-07

**Authors:** John C. Peters

**Affiliations:** New York


### Art. XV.—RELAPSING FEVER IN ANIMALS.

BY JOHN C. PETERS, M.D., NEW YORK.

The official Bengal reports of Veterinary Surgeon, J. H. Steel, and Inspecting Veterinary Surgeon, G. Evans, are models in their way of good work, yea, very good work.

To Dr. Evans belongs the credit of being the pioneer in this new study, but his labors would have been very incomplete without those of Dr. Steel; while it is doubtful if the latter would have advanced as far as he has, without the preliminary work of his predecessor. Dr. Evans made his first discoveries in 1880, while Dr. Steel did not follow until 1884-85.

Dr. Evans first met with the disease in question, in the North-west provinces of India, west of the river Indus, towards the borders of Afghanistan. Shortly before, the 4th Punjaub cavalry had lost 350 horses by this new pestilence ; but as far back as 1876, it had been noticed further west, in Persia. The disease was then generally referred to the use of hard, chop. ped straw for fodder, which scratched and irritated the mouths and throats of the animals ; and to very impure water, the parasites in which, thus got access to the blood. There were irregular attacks of fever, in which the whites of the eyes, and several other mucous membranes became yellow; and petechiæ were also found upon them, especially in the nostrils and vagina. A few days after the animal was put upon the sick list, the fever seemed to subside, and even quite disappear; partial recovery took place, the appetite returned, especially for grass or green fodder; then unexpected relapses would occur, to the number of three or four, and in some cases up to seventeen. The animals were reduced to skeletons; anæmic palpitation of the heart was so severe that the sound could be heard some little distance off. Dropsy was apt to occur, and kidney disease was not uncommon. Some 272 sick horses fell under Dr. Evans' inspection. Then many mules, ninety-eight camels, and some elephants were found with relapsing fever, while bullocks and donkeys escaped. No contagion or inffection was noticed except that which seemed to be carried by a very large brown fly, as big as small bees, and called by a name, which when translated, means, "great needle stings." These flies bit so severely that the blood streamed down the horses' legs as if it had been squirted on.

This gave Dr. Evans a hint, and he introduced blood under the skins of healthy animals, taken from diseased animals by means of hypodermic syringes; he thus reproduced relapsing fever in horses, mules and dogs, and also succeeded when he made these creatures swallow many ounces of blood, drawn from the jugular veins of diseased animals.

He next discovered the parasite which was a *Spirillum* very similar to that which causes relapsing fever in human beings. Next he found that these parasites came and went in successive broods; thus, for a few days, many would be present in every drop of blood, when the fever would run high, the thermometer registering 103 or 105° or even 107°: then they would all pass away, so that only one could be discovered now and then, when the fever would subside, but numberless spores or ova, resembling small granular specks, would be left behind in the blood, to hatch out in a few days more, and occasion a fresh relapse.

Dr. Evans' description of the parasite and its actions, is wonderfully graphic. "It has a blunt head, a tapering body and tail; its motions are undulating and eel-like, and as a rule wonderfully active. Its color is white and difficult to detect, and when dead becomes invisible. It attacks the red globules ferociously, it seizes, worries and shakes them, tears and bites out pieces, so that many red blood globules become shapeless particles of a pale red, jelly-like substance."

Finally Dr. Evans found these or very similar *spirillæ*, in stagnant water and on the root stalks of coarse grasses, and thought that the spores and ova might be conveyed by the winds to distant places and there develop under favorable conditions of heat and moisture.

He went so far as to give the carcasses of dead animals to jackals and pariah, or wild dogs, and thought they conveyed the germs of the disease to drinking water.

Dr. Evans found that the best method to examine the blood was to put a full drop of serum on the slide, lay a thin cover on press it moderately so as to squeeze some of the fluid from under the cover, all around; retain the pressure for a short time, until what has been pressed out dries up, and forms a good temporary cement, which will keep the serum within from drying: the movements of the parasites can then be watched for many hours.

Singularly enough, Dr. Evans did not call this disease a relapsing one, much less a relapsing fever. He did not call much attention to the relapses, and did not compare his *spirillæ* to those which are so well known in the relapsing fevers of men He regarded it as different in important respects, from any other disease ever reported upon, in man or beast: that it was quite new to science, and, as long as the spiral parasite found in the blood was not better known it was advisable to call the disease by the common country name of *surra*, which is an ordinary Afghan name for disease or *rot*, like sheep rot.

THE BURMAH EPIDEMIC.

Late in October, 1884, Inspecting Surgeon J. H. Steel, was ordered from India to Burmah, to investigate an epidemic among the transport mules and animals. He found it evidently not one of the ordinary diseases of the horse; and among the unusual disorders, the only one bearing any analogy to it was *surra*, as described by Dr. Evans, in 1880. Careful and sustaine

*thermometric researches* soon enabled Dr. Steel to prove that it was a true *relapsing fever*, closely resembling, though not absolutely identical with the relapsing fever of man. He then inoculated dogs, horses, mules and monkeys, and reproduced the disease. His first observations were made on fifty-one mules and eleven ponies, out of which he made twenty post-mortem examinations ; finally he had 182 fatal cases, while his horses elephants and bullocks daily diminished in number, and finally the losses among the artillery horses was so great, that the field batteries had to be withdrawn from Burmah. The disease was first decreed to be ulceration of the stomach, which was often found, but that was traced to the use of coarse grass, harsh straw, and unhulled rice, which scratched and tore the enfeebled mucous membranes of the sick animals. The Burmah Veterinary Surgeons had already noticed the high fever and that it recurred, but these occurrences were regarded as separate attacks, not relapses.

Water, from certain foul tanks was suspected of producing the disease, especially as some of the hospital and regimental latrines drained into them, as well as some cemeteries where fever patients had been buried, and near which some of the mules and ponies grazed, In times of freshets and storms, the hospital and regimental privies were flooded, and their contents washed down into some of the stables. These hospitals had had many fever cases in them, doubtless some relapsing fevers, and the privies contained the excretæ of these and other fever cases in large quantities.

Hard usage, scanty, mouldy, coarse and improper food, exposure to incessant tropical rains, and drinking foul water, were supposed by many to be sufficient to account for the disease, but Dr Steel did not think such common causes of feverish attacks were competent to produce so peculiar a disorder. With the thermometer he could always diagnose the cases, and finally a careful watching of the pulse, and heat of mouth, also enabled him to do so : but, of course, the body heat as taken with instruments of precision, was by far the most satisfactory guide. At first the pestilence was regarded as a malarial typhoid affection with attending ulceration of the stomach, instead of the bowels: for Surgeons Steel and Frost had hunted for a month for parasites in the blood, and found none, as they were white and almost transparent. Next they were colored with aniline and magenta dyes, and the whole matter became clear. Dr. Steel also found that the *Spirillum* of relapsing fever in man and animals resembled very closely in form and movements, the *spirillum plicatalis* first described by Ehrenberg as of common occurrence in stagnant water. He too, saw the parasites seize the red blood globules, tear them from their rouleaux, shake them, distort them into various shapes, and show great energy, strength and perseverance in pushing their way through a mass of red corpuscles, and even to bore a passage through the capillaries more easily than leucocytes do.

Numerous inoculation experiments reproduced the disease in four to seven days with an abundance of parasites in the blood. The relapses as marked by the thermometer, occurred about every ten days, lasted several days, and then subsided to recur again. It was found if the thermometer and microscope were used every day for five days, a diagnosis could almost certainly be made out, *if they were used for ten days a positive statement as to the presence or absence of the disorder could be made out, without the possibility of error.*

Enough has perhaps been written for the present. I would merely call attention here to the great similarity between this relapsing disease and the well-known *Beri Beri* of India and Japan. The more carefully the descriptions of the two are studied, the more will it seem that both are varieties of relapsing fever.

Nor is the chapter of the resemblances between human and animal diseases yet closed. In 1881 the first Punjaub Infantry in the northwestern provinces of India, lost in a few weeks over forty men of infectious pneumonia, out of a strength of 550 ; and the fifth Punjaub Infantry lost sixty men. Dr. Costello says that at the time the epidemic appeared among his troops they had just been marching through districts of Afghanistan known to be affected with the cattle plague, called contagious pleuro-pneumonia, from which source the soldiers seemed to have caught the infection.*

It is also equally well known that the great epidemic of

---

*See Wm. Wood Co.'s reprint of Prof. German See's Diseases of the Lungs, p. 64.

cerebro spinal meningitis commenced in this city in a severe outbreak among horses in 1872, fully a year before it appeared among men: and that it has ever since persisted in a mild degree. There have been few signs of infection manifest of this, and some of the other diseases from animals to men, and still fewer of direct contagion. But that all these diseases are communicable in some mysterious way, far more occult than the transmission of typhoid fever, is very evident. Typhoid fever is not at all contagious in the ordinary sense of the word, but that it is one of the most communicable diseases when its poison gets into drains or water courses is well known. Relapsing fever is one of the most contagious diseases in human beings; while among animals its infection seems to sink down to a plane comparable to that of typhoid fever. Horse influenza will run through the whole of a large stable if a single contaminated animal is shut up in it over night; but it does not affect the stableman, except rarely. Human influenza is an exceedingly communicable disease to other human beings, but does not seem to affect animals. Up to lately the pleuro-pneumonia of cows has not seemed communicable to human beings, but we do not know what ravages it may have caused among delicate persons. A few years or merely months ago, any one who had spoken seriously about infectious pneumonia and consumption among human beings would have been hooted at: now every one believes in them. The whole doctrine of infectious contagion will have to be rewritten in the near future.

